# Traditional Chinese medicine on treating epididymitis: A systematic review and meta-analysis protocol

**DOI:** 10.1097/MD.0000000000015975

**Published:** 2019-06-14

**Authors:** Yi Lei, Jie Yang, Yongqiang Li, Xudong Yu, Sheng Deng, Chaohui Xue, Wei Zheng, Jianwei Shang, Yahui Xue

**Affiliations:** a Department of Andrology, The Second Affiliated Hospital of Shaanxi University of Traditional Chinese Medicine, Shaanxi; b Beijing Fengtai Maternal & Child Health Hospital; c Department of Andrology, Dongzhimen Hospital, Dongcheng District, Beijing, China.

**Keywords:** epididymitis, protocol, systematic review, traditional Chinese medicine

## Abstract

**Background:**

Epididymitis is a common disease in nonspecific infections of the male reproductive system according to the clinical incidence of acute epididymitis and chronic epididymitis. Many clinical trials have proven that Chinese medicine has a significant effect in the treatment of epididymitis. In this systematic review, we aim to evaluate the effectiveness and safety of traditional Chinese medicine (TCM) for epididymitis.

**Methods:**

We will search for PubMed, Cochrane Library, AMED, Embase, WorldSciNet, Nature, Science online, China Journal Full-text Database (CNKI), China Biomedical Literature CD-ROM Database (CBM), and related randomized controlled trials included in the China Resources Database. The time is limited from the construction of the library to November 2018. We will use the criteria provided by Cochrane 5.1.0 for quality assessment and risk assessment of the included studies, and use the Revman 5.3 and Stata13.0 software for meta-analysis of the effectiveness, recurrence rate, and symptom scores of epididymitis.

**Ethics and dissemination:**

This systematic review will evaluate the efficacy and safety of TCM for treating epididymitis. Because all of the data used in this systematic review and meta-analysis has been published, this review does not require ethical approval. Furthermore, all data will be analyzed anonymously during the review process.

Registration number: PROSPERO CRD42019130569

## 1 Introduction

Epididymitis, which is a common disease in nonspecific infections of the male reproductive system, is found in male of all ages, but most of them are young adults aged 20 to 40 years, which accounting for 70% of all the patients who suffer from it.^[1,2]^ It is, according to the clinical incidents, classified into 2 types such as acute epididymitis and chronic epididymitis, and the chronic type is more common. The main clinical manifestations contain referred pain in hypogastrium and groin, enlarged scrotum, increased pain when standing or walking.^[3]^ During acute phase, the clinical features such as local redness and pain, sudden rise in temperature, and leukocytosis will be manifested. Besides, nodules or lumps or turning hard could be detected through physical examination, accompanied with mild or moderate tenderness, and the vasodilatation of vas deferens could also be detected on the pathologic side.^[4]^ The cause of epididymitis is complicated and varied, but the pathogenesis of the disease is usually identified as secondary to prostatitis or urinary tract infections. The disease has been seriously affecting patients’ mental health and quality of life.^[5]^ Meanwhile, it could also reduce the production and development of sperm because of the various degree of the inflammation in the epididymis, which resulting in a significant increase in sperm deformity; therefore, the risk of suffering from infertility is increased.^[6–8]^

Currently, definite therapy for epididymitis has not been settled, and the use of antibiotics and anti-inflammatory drugs are still the mainly treatment.^[9]^ But for the recurrent chronic epididymitis, the drugs could not be easily effective because of the susceptibility of nodules.^[10,11]^ Besides, the chronic type could suffer from varicocele easily and the local congestion would be formed naturally, which lessens the local immunity and leads to the lingering disease more easily.^[12,13]^ Aiming at this situation, a few patients accept the therapy of epididymectomy which, however, does not enhance the overall efficacy because of the deficiency of the quality of sexual life.^[14,15]^

Traditional Chinese medicine (TCM) has been widely used in clinical trials in recent years. It has been undoubted that TCM has obvious curative effect in reducing chronic pain in the epididymis, reducing scrotal swelling and anti-tissue fibrosis, which have been shown by recent studies.^[16]^ The studies also have shown that a specific ingredient in Chinese herbal medicine could achieve the desired results of peripheral analgesia, which mainly by the means of adjusting body function to accelerate the production of endogenous opioid peptides in the central nervous system and activate the analgesic system in the body of the patient.^[17,18]^ Besides, it could also achieve anti-inflammatory effects by increasing the levels of β-Ep in inflammatory tissues and serum.^[19,20]^ According to the theory of TCM, we believe that it could regulate the balance of qi and blood in human body, which ameliorates the function of the human body by stimulating the regulated acupoints. This kind of therapy has been increasingly popular among doctors and patients because of the unique advantages of simplicity, convenience, efficacy, and low cost.

In the preliminary searches of the electronic databases, we found that randomized controlled trials (RCTs) of TCM for epididymitis are on the rise.^[21,22]^ However, due to the limitation of the size and number of clinical centers, most clinical trials are small samples with low quality and lack of evidence-based exploration. Besides, the publication of the similar systematic review has not been retrieved in the database. Therefore, this review of epididymitis adopts meta-analysis to evaluate the efficacy and safety of TCM in the treatment of epididymitis and provides evidence for its application in clinical practice.

## 2 Methods

This systematic review protocol has been registered on PROSPERO CRD42019130569 (http://www.crd.york.ac.uk/PROSPERO/display_record.php?ID=CRD42019130569). The protocol follows the Cochrane Handbook for Systematic Reviews of Interventions and the Preferred Reporting Items for Systematic Reviews and Meta-Analysis Protocol (PRISMA-P) statement guidelines. We will describe the changes in our full review if needed.

### 2.1 Inclusion criteria for study selection

#### 2.1.1 Types of studies

We will gather all studies of TCM therapy in treating epididymitis, no matter whether they have been published or not, based on the method of RCT. The language is limited to Chinese and English. Non-RCTs quasi-RCTs, series of case reports, and cross research will be excluded.

#### 2.1.2 Types of participants

Male patients who have been diagnosed with epididymitis will be included, which means that there are no limitation in age, regional, national, ethnic, and sources.

#### 2.1.3 Types of interventions

The drug composition, the dose-sepididymitiscific Chinese medicine preparation, or the combined western medicine are used as exepididymitisrimental interventions. Both prescription and Chinese patent medicines will be included. Other TCM treatments such as intravenous medication, TCM, and massage will be limited.

#### 2.1.4 Control interventions

As for the control interventions, who accepted simple western medicine can be used as a control intervention or did not get any treatment as a blank control would be adopted. However, once they had accepted the therapy of TCM, the trials will be rejected.

#### 2.1.5 Types of outcome measures

##### 2.1.5.1 Primary outcomes

The main criterion is epididymal color Doppler ultrasonography.

##### 2.1.5.2 Secondary outcomes

Secondary evaluation criteria included that whether there were amelioration of the symptoms of patients, tenderness in epididymis, recovery in size, and changes in safety indicators (blood routine urine routine liver and kidney function). Meanwhile, whether there were occurrences of adverse reactions or adverse events during the experiment should be paid close attention to comprehensively evaluate the clinical efficacy and safety of TCM for epididymitis.

### 2.2 Search methods for the identification of studies

#### 2.2.1 Electronic searches

Database Search: Search PubMed, Cochrane, Library, AMED, Embase, WorldSciNet, Nature Science online, China National Knowledge Infrastructure(CNKI), and China Biology Medicine disc (CBMdisc). The temporal interval is limited from the time that the databases created to May 2019, and the combination of keyword and free word retrieval is adopted. The search terms include “Chinese medicine,” “traditional Chinese medicine,” “proprietary Chinese medicine,” “Chinese herbal medicine,” “ epididymitis,” and “sub-sputum.” The search term in the Chinese database is the translation of the above word. The complete PubMed search strategy is summarized in Table 1.

**Table 1:**
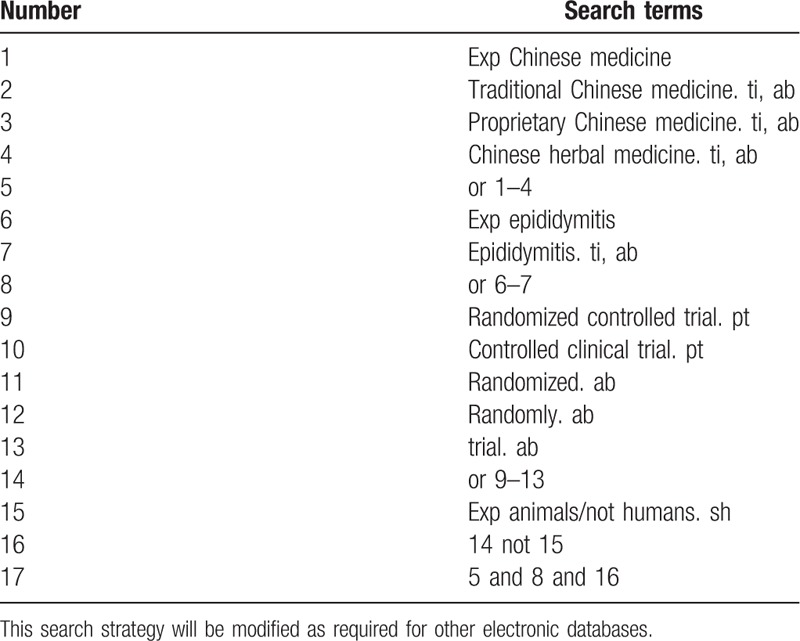
Search strategy used in PubMed database.

#### 2.2.2 Searching other resources

The manual search mainly searched for relevant literatures, earlier than the database above-mentioned, such as “China Rehabilitation Medicine Journal,” “Chinese TCM,” “Chinese Journal of Physical Medicine and Rehabilitation,” “TCM Clinical Journal,” and “Chinese Journal of Urology.”

### 2.3 Data collection and analysis

#### 2.3.1 Study identification

**Figure 1:**
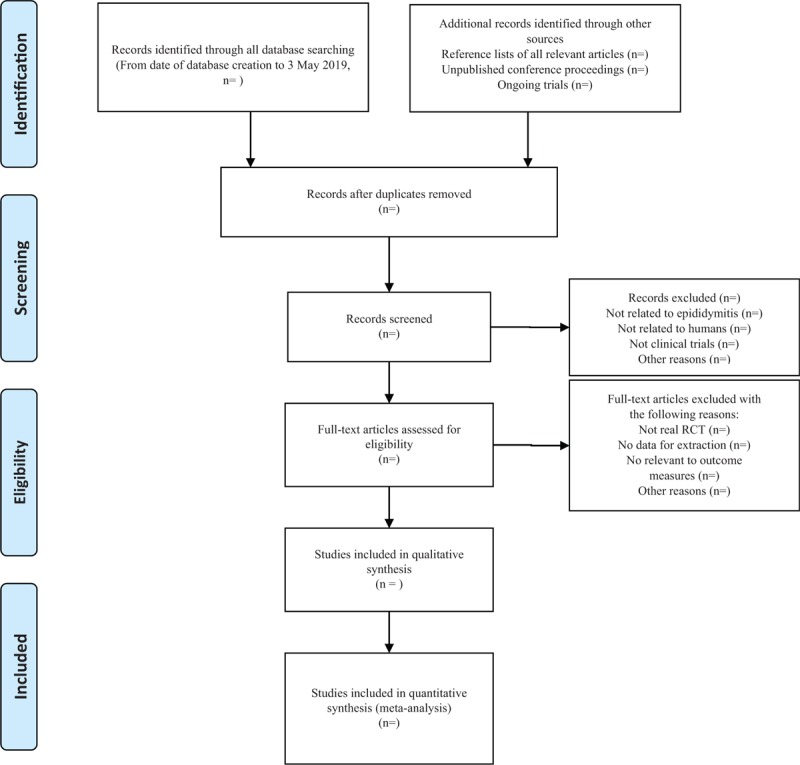
The PRISMA flow chart.

1. There are 2 researchers filtering out the literature that clearly do not conform to the study such as meeting minutes dissertations reviews animal experiments and so on, which, after excluding all the retrieved documents from the duplicated literature, adopt the method of reading the title of the literature abstracts, etc. The details of selection process are shown in the PRISMA flow chart in Figure 1.
2. The 2nd time of screening the literature: Skimming the remaining documents and filtering out unqualified documents such as case reports, theoretical discussions, and nonconformance of interventions.
3. The 3rd time of screening the literature: Carefully reading the remaining documents and strictly filtering out unqualified documents such as general controlled trials, lacking control group, deficiency of random allocation, incompatible outcome indicator, the appearance of similar data, etc.
4. As for the literature that cannot be ensured, it would be confirmed by the discussion of the 2 researchers. And if they cannot reach an agreement, the 3rd-party experts would get involved, which aims at absorbing the appropriate RCTs into the study.

#### 2.3.2 Data extraction and management

The literature data extraction will be completed independently by 2 researchers and the data form uniformly developed by the researcher was filled out. The data extraction content includes the following:

1. General information: article title, 1st author, corresponding author, time of publication research, evaluation correspondence, contact information.
2. Research method: design pattern, ample size, random allocation, random hiding, blind method, baseline level.
3. Participants: patient's age, gender, epididymitis diagnostic criteria, severity, ethnicity study, location.
4. Intervention: TCM, TCM point, period of treatment, treatment frequency
5. Efficacy evaluation: Main observation indicators secondary observation indicators safety indicators and number of adverse reactions.
6. Note: sources of funds, medical ethics audit, important references.

#### 2.3.3 Assessment of risk of bias in included studies

As for the literature quality evaluation, we will use the bias risk assessment tool recommended by Cochrane to assess the quality of all included literature and risk of bias. The assessment includes: sequence generation; allocation concealment; blinding of participants, personnel, and outcome assessors; incomplete outcome data; selective outcome reporting; and other sources of bias. The evaluation above would be independently evaluated by 2 researchers. If there are different opinions, we discuss them. If there are still differences exist, we would consult the third appraiser. Otherwise, we need to consult with the Cochrane Professional Group for solution.

#### 2.3.4 Statistical analysis

The meta-analysis studied in this review will adopt Rev Man 5.3 and Stata 13.0 statistical software. Heterogeneity test will be used for the inclusion of the study, and random or fixed effect models will be adopted, with *P* < .05 as the test standard. If the heterogeneity between the results is too large, the random effects model, which deduce the source of heterogeneity by sensitivity analysis, will be used for the rest analysis. Secondly, according to the different type of statistical data, the binary categorical variable will use the odds ratio and its 95% confidence interval (CI) as the effect analysis index. As for the continuous variable, the standardized mean difference and its 95% CI will be used as the effect analysis index. If the outcome measures only provide the means and standards deviation before or after treatment, the Mean_change_ and the SD_change_ are obtained according to the method provided in Cochrane Handbook 5.1.0: 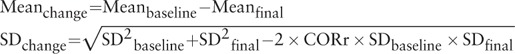


The forest map and funnel plot were drawn and analyzed using Rev Man 5.3 software, and the funnel plot was used to analyze potential publication bias. As for the literature quality evaluation, we will use the bias risk assessment tool recommended by Cochrane to assess the quality of all included literature and risk of bias. The assessment includes: sequence generation; allocation concealment; blinding of participants, personnel and outcome assessors; incomplete outcome data; selective outcome reporting; and other sources of bias. The evaluation above would be independently evaluated by 2 researchers. If there are different opinions, we discuss them. If there are still differences exist, we would consult the 3rd appraiser. Otherwise, we need to consult with the Cochrane Professional Group for solution.

#### 2.3.5 Publication bias

If a result of a meta-analysis contains more than 10 articles, we will use a funnel plot to test the risk of publication bias.

#### 2.3.6 Quality of evidence

The quality of evidence for the main outcomes will also be assessed with the Grading of Recommendations Assessment, Development and Evaluation approach. The evaluation included bias risk, heterogeneity, indirectness, imprecision, and publication bias. And each level of evidence will be made “very low,” “low,” erate,” or “high” judgment.

## 3 Discussion

In recent years, the clinical RCTs about epididymitis have been increasing continuously; however, it is still unsatisfactory in the diagnosis and therapy of the disease.^[15]^ The clinicians have not reached a consensus on the therapeutic principles and evaluations of the disease, and lack unified normalized standards. At present, there is no large-scale epidemiological investigation on this disease, and there are few reports in related literatures. TCM has a profound theoretical foundation and rich clinical experience in the treatment of chronic epididymitis.^[21,22]^ TCM, which an essential part of TCM possesses the characteristics of small side effects and simple and easy operation, has long been used to treat genitourinary diseases such as prostatitis.^[23,24]^ This kind of therapy mainly achieves therapeutic effects by stimulating the body's righteousness and regulating the balance of qi and blood, besides, yin, and yang.^[25]^ Although the specific mechanism of TCM treatment of epididymitis is not very clear, clinical studies have shown that TCM treatment of epididymitis can relieve pain and improve symptoms to some extent.^[16]^ As far as we know, there has not had any comparison of the effectiveness of TCM in the treatment of epididymitis. Therefore, we will use systematic review and meta-analysis to evaluate the efficacy and safety of TCM for the treatment of epididymitis. The results of this study can provide a possible ranking for TCM treatment of epididymitis. In addition, the quality of evidence for the primary outcome will be assessed using a scoring method. We hope that these results will provide clinicians with the basis for TCM treatment of epididymitis and provide the best choice for the treatment of patients. In addition, although this study will conduct a comprehensive search, it will not search for languages other than Chinese and English, which will lead to some bias.

## Author contributions

Data curation: YL, JY, SD

Formal analysis: YL, JY, XDY

Funding acquisition: JWS

Project administration: JWS, YHX

Supervision: YQL, YHX

Validation: SJW

Writing – original draft: YL, JY, CHX

Writing – review & editing: JSW, WZ, CHX

**Conceptualization:** Yahui Xue.

**Data curation:** Wei Zheng, Jianwei Shang.

**Formal analysis:** Sheng Deng.

**Funding acquisition:** Jianwei Shang, Yahui Xue.

**Investigation:** Xudong Yu.

**Project administration:** Chaohui Xue.

**Resources:** Yongqiang Li, Xudong Yu, Chaohui Xue.

**Software:** Yongqiang Li, Xudong Yu, Wei Zheng.

**Supervision:** Yi Lei, Chaohui Xue, Wei Zheng.

**Validation:** Jie Yang, Yongqiang Li.

**Visualization:** Sheng Deng.

**Writing – original draft:** Yi Lei, Jie Yang, Chaohui Xue, Jianwei Shang, Yahui Xue.

**Writing – review & editing:** Chaohui Xue.
